# High Frequency, Spontaneous *motA* Mutations in *Campylobacter jejuni* Strain 81-176

**DOI:** 10.1371/journal.pone.0088043

**Published:** 2014-02-18

**Authors:** Krystle L. Mohawk, Frédéric Poly, Jason W. Sahl, David A. Rasko, Patricia Guerry

**Affiliations:** 1 Enteric Diseases Department, Naval Medical Research Center, Silver Spring, Maryland, United States of America; 2 Institute for Genome Sciences, Department of Microbiology & Immunology, University of Maryland School of Medicine, Baltimore, Maryland, United States of America; Iowa State University, United States of America

## Abstract

*Campylobacter jejuni* is an important cause of bacterial diarrhea worldwide. The pathogenesis of *C. jejuni* is poorly understood and complicated by phase variation of multiple surface structures including lipooligosaccharide, capsule, and flagellum. When *C. jejuni* strain 81-176 was plated on blood agar for single colonies, the presence of translucent, non-motile colonial variants was noted among the majority of opaque, motile colonies. High-throughput genomic sequencing of two flagellated translucent and two opaque variants as well as the parent strain revealed multiple genetic changes compared to the published genome. However, the only mutated open reading frame common between the two translucent variants and absent from the opaque variants and the parent was *motA*, encoding a flagellar motor protein. A total of 18 spontaneous *motA* mutations were found that mapped to four distinct sites in the gene, with only one class of mutation present in a phase variable region. This study exemplifies the mutative/adaptive properties of *C. jejuni* and demonstrates additional variability in *C. jejuni* beyond phase variation.

## Introduction

*Campylobacter* is one of the most common sources of diarrheal disease in developing as well as developed countries [1]. In the United States, *Campylobacter* is the second leading cause of foodborne illness among pathogens monitored by the Foodborne Diseases Active Surveillance Network, or FoodNet [2]. Infection with *Campylobacter*, or campylobacteriosis, is a self-limiting disease associated with a variety of symptoms that range from watery diarrhea to dysentery accompanied with fever. In addition, *Campylobacter jejuni* infection has been linked to the development of post-infectious sequelae including Guillain-Barré syndrome [3], [4], reactive arthritis [3], and irritable bowel syndrome [5]–[7]. Associations with inflammatory bowel disease [8], [9] and Celiac disease [10] have also been suggested.

The first published *C. jejuni* genome sequence, that of strain NCTC11168, revealed reversible phase variation in genes encoding surface antigens mediated by slip strand mismatch (SSM) repair at homopolymeric tracts of 8 or more Gs or Cs [11]. Many of these GC tracts are located in genes involved in the biosynthesis of surface structures including the polysaccharide capsule and lipooligosaccharide (LOS), both of which have been demonstrated to be phase variable [12]. Phase variation has also been reported to affect shorter poly-A tracts located in *flgR* and *flgS*, a two-component system that regulates expression of *C. jejuni* flagella [13], [14]. Thus, it is thought that phase variation is a mechanism whereby the bacteria can modify the antigenic make-up of its surface to evade the host immune system or adapt to new hosts or environments. The frequency of phase variation in *C. jejuni* ranges from approximately 1×10^−3^ to 4×10^−4^ mutations/division and is dependent on the length of the homopolymeric tract [15]. SSM errors are attributed to the absence of a functional methyl-directed DNA mismatch repair system (MMR) in *C. jejuni* [11].

Strain 81-176, first isolated during a 1981 outbreak of acute enteritis in Minnesota associated with consumption of contaminated raw milk [16], is one of the best characterized strains of *C. jejuni*. Strain 81-176 has been demonstrated to invade human epithelial cell lines at high levels *in vitro* [17], [18], to be virulent in human [19], [20] and primate models of diarrheal disease [21], [22], to be amenable to genetic analysis, and has been sequenced by two independent groups [23], [24]. Here we describe colonial variation of *C. jejuni* strain 81-176 that is associated with loss of motility. Sequence comparison of five derivatives of strain 81-176 from the same lineage revealed several changes from the published genome [24]. Among the five derivatives sequenced were two non-motile, translucent variants, both of which were mutated in *motA*, a gene necessary for energy transmission to the flagellum and subsequent motility. Further characterization of the translucent phenotype indicated a general association with loss of motility. Random sequencing of the *motA* gene of 56 additional translucent variants indicated that 29% of these were *motA* mutations and that the mutations occurred at 4 distinct sites in the gene. These included SSM at a homopolymeric tract, a deletion, an insertion, and a transversion. Collectively, the data underscore the genomic instability of *C. jejuni*.

## Results

### Appearance of colonial variants

**Figure 1:**
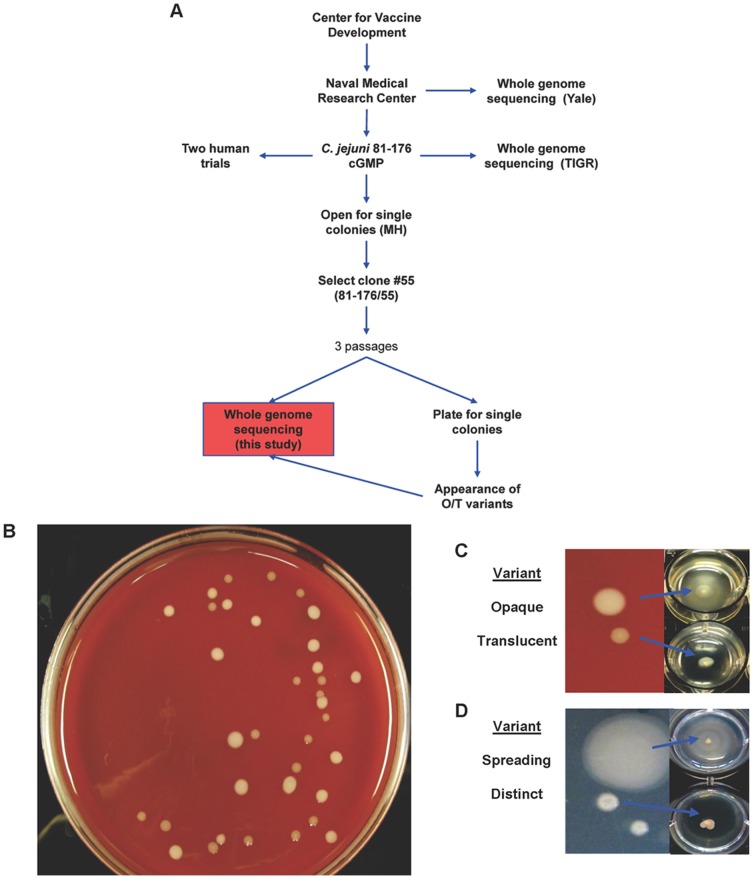
Derivation of 81-176/55 and the appearance of colonial variants linked with motility. (A) Cartoon depicting the origin of the sequenced strains. Abbreviations include O for opaque and T for translucent. (B) Appearance of colonial variants of *C. jejuni* 81-176 grown on CBA plates in microaerobic conditions following dilution and plating. Colony morphology was examined on CBA (C) as well as MH agar (D) and motility analysis made use of 0.6% BB agar 24-well plates with single colonies stabbed into the wells. (E) Transmission electron microscopy images of negatively stained WT, opaque, and translucent variants of *C. jejuni* strain 81-176.

The lineages of 81-176 are shown in Figure 1A. Strain 81-176/55 was isolated as single, encapsulated colony from the cGMP-stock of 81-176 that was sequenced by The Institute for Genomic Research (TIGR) [24]. When strain 81-176/55 was plated for single colonies on Campylobacter Blood Agar (CBA), two distinct colonial variants were observed (Figure 1B). These variants were opaque (O), appearing as a slightly larger light/white colonies, or translucent (T) colonies having a darker hue. The frequency with which the T variants appeared amongst the population of strain 81-176/55 was on average 17% (range 0–50%). Given that colonial variation in *C. coli* has been linked with motility [26], variant colonies were tested in soft agar (Figure 1C). While the O colonies were motile, the three T colonies tested were non-motile and lacked darting motility in wet mounts. Motility is also known to be phase variable [13], [14] and thus expression of FlgR, FlgS, and flagellin was examined by immunoblot in the T variants (data not shown; [25]). All three colonies expressed FlgR, FlgS, and flagellin, and appeared flagellated by transmission electron microscopy (Figure 1E). Several genes known to be associated with a paralyzed flagella filament (*pflA, flgP, flgQ*) were PCR-amplified and sequenced, but no sequence changes were evident (data not shown).

### Whole genome sequencing

**Figure 2:**
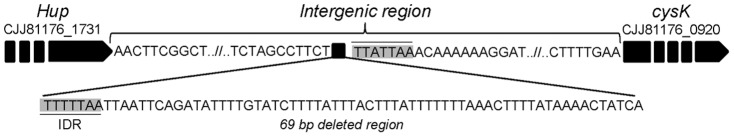
Schematic illustration of the 69-176/55 and its 4 additional sequenced offspring. The assembly of the whole genome sequences revealed varying SNPs throughout the chromosome. One confirmed difference between the previously sequenced 81-176 and 81-176/55 (as well as the opaque and translucent progeny) was the presence of a 69 bp deletion in the intergenic region between *hup* and *cysK*. An incomplete direct repeat (IDR) bracketing the deletion is indicated.

In an effort to identify the genotypic cause of the non-motile, flagellated variants, two O (O1 and O3) and two T (T1 and T3) variant progeny, as well as the 81-176/55 parent, were sequenced using Illumina technology (Figure 1A). Alignment of the 81-176/55 parent sequence to the published sequence [24] also allowed comparison of two lineages of 81-176 with relatively few passages between them. When the sequences were assembled and compared to the published reference 81-176 sequence [24], varying numbers of mutations were revealed throughout the genome. Beyond the expected variation in homopolymeric tracts, single nucleotide polymorphisms (SNPs) and indels (short insertions or deletions) located mostly in intergenic regions were identified (Table S1). One of these variations was identified between CJJ81176_0920 (encoding CysK, cysteine A biosynthesis protein) and CJJ81176_1731 (encoding hup, a histone-like DNA-binding protein). Conventional sequencing confirmed a deletion of 69 bp in the intergenic region in front of CJJ81176_0920 (*cysK*; Figure 2). This deletion, while present in 81-176/55 and all four variant offspring, was not observed in the 81-176 reference sequence or the whole genome sequencing performed by Hofreuter *et al*. [23]. The deletion may have occurred between an imperfect direct repeat of 7 bp (Figure 2).

**Figure 3:**
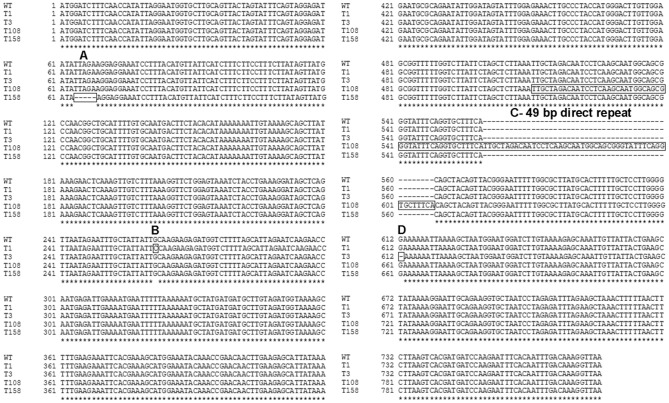
Nucleotide alignment of *motA* and mutated alleles. The four mutations found within *motA* have been assigned a type for ease in distinguishing. Type A mutation is a deletion at base 64 (T158). Type B mutation is a missense mutation at base 262 (T1). Type C mutation is a duplication of 49 bp (boxed) creating a direct repeat (T108). Type D mutation is a nonsense mutation at base 612 (T3).

**Figure 4:**
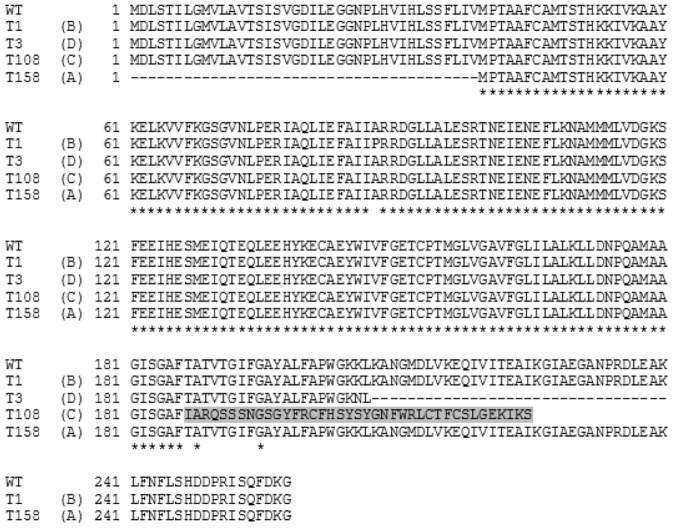
Amino acid alignment of MotA. The *motA* mutations are predicted to result in non-functional proteins. A missense mutation at base 262 (T1, Type B) resulted in an A to P mutation at amino acid 88; a nonsense mutation at base 612 (T3, Type D) resulted in a premature truncation; a duplication of 49 bp created a direct repeat within *motA* and led to the replacement of the last 73 residues in the C-terminus with a sequence of 39 new residues that are highlighted (T108, Type C); and a deletion at base 64 resulting in a truncated protein (T158, Type A).

The only mutated open reading frame common to both sequenced T variants but absent from the O variants, 81-176/55, and the two published 81-176 sequences was CJJ81176_0359, encoding the flagellar motor protein MotA. Interestingly, the two sequenced T variants each had distinct mutations in *motA*. Variant T1 had a G to C transversion at base pair 262 (labeled B in Figure 3) that resulted in an A87P mutation (Figure 4). Variant T3 had a SNP at base 612 that resulted in a G5 to G4 tract change (labeled D in Figure 3) and led to a truncation of 53 amino acid residues in the C-terminus of MotA (Figure 4).

### Association of the T phenotype with loss of motility

To further confirm the role of *motA* mutation in the appearance of the T variants, a *motA* insertional inactivation mutant was generated and shown to have a ‘translucent’ phenotype comparable to what was seen for the spontaneous T variants (data not shown). However, insertional mutants in flagellin and *pflA* also displayed the T phenotype (data not shown). Thus, the T phenotype appeared associated with loss of motility by a variety of mutations, and the two that were picked for genomic sequencing happened to both contain a mutated *motA* gene.

### Colonial variation on other media and by other lineages of 81-176

To determine if O/T variation was specific to CBA plates, 81-176/55 was plated for single colonies on Mueller-Hinton (MH) agar. Instead of O and T variation, distinct and spreading colonies were observed (Figure 1D), similar to what has been previously described for *Campylobacter* [26]–[28]. Furthermore, when plated to MH, the T variants isolated on CBA appeared as distinct colonies and the O variants appeared as spreading colonies. Thus, for simplicity, all colonial variants having either a translucent or distinct phenotype will be referred to as T whereas the spreading and opaque variants will be referred to as O. Colonial variation was not unique to 81-176/55 as a variety of 81-176 lineages, including cGMP 81-176 (the parent strain of clone #55) [20] and 6 laboratory stocks frozen at different times, were also observed to form T and O colonies. Overall, the T variants occurred amid a population at a frequency of ∼20% regardless of lineage.

### Incidence of *flgR* and/or *flgS* mutants among T variants

Since *flgR* and *flgS*, encoding a two component system regulating flagella expression, are phase variable [13], [14], we examined twenty non-motile T variants to determine expression of FlgR and/or FlgS by immunoblotting [25]. The results indicated that three colonies (15%) were FlgR^−^ FlgS^+^, three colonies (15%) were FlgR^+^ FlgS^−^, 10 colonies (50%) were FlgR^−^FlgS^−^, and only 4 colonies (20%) were FlgR^+^ FlgS^+^.

### Sequencing of *motA* in additional T variants

Non-motile T variants from different lineages of strain 81-176 were isolated from blood agar, MH, or Blaser-Wang (BW). The *motA* gene was PCR amplified and sequenced from a total of 56 individual non-motile colonies, each from independent platings to avoid characterization of sibling colonies. Most of the colonies sequenced expressed a wildtype *motA* gene (40/56 or 71%) and were presumably mutated in *flgR* and/or *flgS*. A total of 16 colonies (29%) were *motA* mutants and the mutations observed fell into four classes, two of which had been observed in the two original T variants. The four classes were called Type A, B, C, or D (Figure 3 and Table 1). Eight additional variants exhibited the G5 to G4 change exemplified by the T3 mutant (Type D mutation). Four additional variants had an A87P change similar to the previously sequenced T1 mutant (Type B mutation). The third mutation observed occurred in three variants and was found to be a duplication of 49 bp that created a direct repeat within *motA* (Type C mutation, represented in the T108 mutant). The consequence of the duplication event was to replace the last 73 residues in the C-terminus with a sequence of 39 new residues (Figure 4). Finally, a single variant, T158, with a deletion of five nucleotides at base pairs 64-68 (Type A mutation), was found that created a nonsense mutation likely resulting in the use of an alternative start codon and the generation of a truncated protein (Figure 4).

**Table 1:** Sequenced T (translucent or distinct) variants with a *motA* mutation listed by type (see Figure 3).

Type	Mutation	Position	Sequence	Representative	# of Variants (%^1^)	Rev^2^
A	Deletion	64	TTAGA	T158	1 (5)	N/D
B	SNP	262	G→C (A→P)	T1	5 (28)	Yes
C	Insertion	559	49 bp	T108	3 (17)	Yes
D	Deletion	612	G tract	T3	9 (50)	Yes

1 Percentage of variants out of 18 total.

2 Reversion of the mutation confirmed by sequence analysis. N/D indicates that analysis was not done for this single variant.

Representative mutants of Type B (T1), Type C (T108), and Type D (T3) were incubated in semi-soft motility agar (Table 1) to determine if the mutations were revertable; the single Type A mutant was unavailable for testing. Motile forms of each mutant were isolated from the motility agar and sequence analysis of the *motA* gene of each demonstrated restoration of the wildtype sequence.

## Discussion

Phase variation is thought to provide *C. jejuni* an adaptive advantage, aiding in virulence and/or survival. The high degree of genomic variability exhibited by *C. jejuni* isolates results from horizontal gene transfer, intragenomic rearrangements, and the presence of homopolymeric repeats within the genome [29]. Stretches of nucleotide repeats prone to mispairing during DNA replication have been described to abound within the genome of *C. jejuni* [11], [13] and the frequency of variation due to SSM is known to vary based on the length of the repetitive unit [15]. *C. jejuni* lacks the prototypical MMR of *E. coli* [11], [24] which functions to correct single base mismatches or short nucleotide runs that escape proofreading during DNA replication [30]. A defect in MMR has been demonstrated to contribute to SSM [31], [32] and a hypermutator phenotype in a number of bacteria [31], [33]–[35]. Thus, it is likely that the absence of MMR facilitates the genetic diversity seen in *C. jejuni*.

Indeed, *C. jejuni* has been suggested to be unstable in culture. Strain NCTC11168 has varying phenotypes; different lineages display transcriptional differences and varying virulence capabilities *in vitro* and *in vivo* [36]–[39]. Re-sequencing of NCTC11168 lineages and comparison to NCTC11168-GS, the original sequence generated in 2000 [11], revealed extensive variation in contingency loci with generally fewer SNPs/indels outside these regions [38]–[40]. Re-sequencing following mouse-adaptation [38] or human infection [41] also revealed changes primarily within contingency genes.

Strain 81-176 has been previously sequenced by two independent groups, both of which received the strain from the NMRC (Figure 1A). The first whole genome sequencing, by Hofreuter *et al.* at Yale University, was performed by high-throughput sequencing using 454 sequencing technology [23]. The second whole genome sequencing was performed by conventional dideoxy sequencing at TIGR [24]. Both strains were derived from the same stock of 81-176, but were of different lineages (Figure 1A). Although no comparison of the two sequences has been published, a direct alignment revealed many single nucleotide deletions. Many of these differences are likely due to the different technologies used for sequencing. Here we had the opportunity to compare five related isolates of 81-176–two T variants, two O variants, and the parent of both–using the same sequencing technology. Despite minimal passage of the strains, sequence variation was evident as shown in Table S1. 81-176/55 and all derivatives had a 69 bp deletion in the intergenic region in front of CJJ81176_0920 compared to the TIGR sequence. This deletion was not present in the 81-176 strain sequenced at Yale, which was derived from the NMRC laboratory stock. The 69 bp deletion most likely affects the transcriptional start site (TSS) of *cysK*. CysK, the enzyme encoded by CJJ81176_0920, is involved in the biosynthesis of L-cysteine from L-serine. It remains unclear as to how this deletion happened, but an imperfect direct repeat (TTTTTAA and TTATTAA) of seven base pairs flanks the deleted region (Figure 2).

In the current study, the presence of translucent, non-motile variants of 81-176/55 was detected in a population of opaque, motile bacteria. Although the two original T variants sequenced were *motA* mutants, the non-motile population was composed of both flagellated and non-flagellated forms. Random sequencing of the *motA* gene of 56 translucent colonies revealed that 16 (29%) were mutated in this gene. Thus, a total of 18 independent spontaneous *motA* mutations were sequenced and the mutants fell into 4 distinct classes, only one of which could be considered classical SSM at a homopolymeric tract. Interestingly, motile revertants to 3 of these variant classes were readily detected by plating onto semi-soft motility agar; the only representative with a type A mutation was not tested.

In this study we demonstrate a link between colonial variation and motility in strain 81-176. It remains to be determined if similar variations occur in other *C. jejuni* strains. Although phase variation of motility [13], [42] and colonial variation of *C. jejuni* [26], [27] have both been described, an association between colony morphology and motility in *C. jejuni* has not, to the best of our knowledge, been reported previously. In the related species *C. coli*, Park *et al.* reported on the appearance of large, swarming colonies and smaller, pin-point colonies [28]. The different colony morphologies of *C. coli* were linked to variation from a flagellated to non-flagellated form due to phase variation of a homopolymeric T tract in *flhA*, a tract not found in the *flhA* gene of *C. jejuni* [13].

Hendrixson previously characterized multiple spontaneous changes in *flgS* using a genetic screen [14] and the breadth of changes described here for *motA* are reminiscent of those described for *flgS*. In *flgS*, the deletion of a duplicated ACCTT run resulted in premature termination of the protein. Here, a deletion of a TTAGA near the 5′ end of *motA* (Type A mutation) resulted in a nonsense mutation the consequence of which is likely the use of an alternative start codon truncating the protein. Additionally, the duplication of a 49 bp sequence in *motA* (Type C mutation) resulted in premature termination of the protein. Indeed, mutation of *motA* occurred by a variety of mechanisms only one of which, the SNP at base 612 that results in the deletion of a single guanine nucleotide within a homopolymeric tract (Type D), is similar to classical phase variation described to occur via infrequent homopolymeric G tracts located within the AT-rich genome [11], [13], [14].

MotA and MotB form the stator complex of the flagella that provides energy to the flagellar motor by conducting the flow of protons [43]. The 258 residue MotA protein shares 24% (56/234) identity and 44% (104/234) similarity with the *E.coli* counterpart (Figure S1A). Prediction of transmembrane helices [44] allowed identification of a similar conformation for *C. jejuni* MotA in comparison to the *E.coli* protein (Figure S1B). Both proteins contain two inner membrane domains with two large cytoplasmic domains. Based on comparison with the MotA of *E. coli*, predictions can be made regarding the effect of the 4 mutations within *motA* on *C. jejuni* protein function. Both Type C and Type D nonsense mutations result in premature protein termination; the deletion of the C-terminus region of MotA in these mutants is likely to render the protein non-functional as this region is localized in the cytoplasm and is crucial for torque generation [43]. An additional nonsense mutant, Type A mutation, occurs early in translation likely resulting in the use of alternative start codons and production of a truncated protein sequence missing the N-terminus 39 amino acids. The remaining mutation, Type B, is a missense mutation whereby a SNP confers an A87P change. Mutational studies in *E. coli* have demonstrated that addition or deletion of proline residues in MotA was detrimental to torque generation [43]. In particular, four residues of MotA were critical for its functionality: Arg90, Glu98 (cytoplasmic domain), Pro173, and Pro222 (interface between the cytoplasmic domain and the membrane domain). Studies in different bacterial species point out the role of charged residues (Glu and Arg) in the function of the stator as well as the role of proline in the conservation of the conformation [45]. As proline residues have a crucial role in keeping the conformation of MotA optimal [43], the introduction of an additional proline in Type B mutants most likely disturbs the final conformation of the protein rendering it non-functional.

The ability to phase vary flagellar expression by SSM of *flgR/S* reduces the energy required to synthesize the filament, but an advantage to high frequency mutation of a motor gene in a bacterium expressing a full-length filament is not obvious. *C. jejuni* is known to secrete a variety of non-flagellar proteins through the flagellar filament in the absence of a specialized type three secretion system, some of which are thought to play a role in virulence or commensalism [46]–[49]. A *motA* mutant retained the capacity to secrete at least one of these proteins, FspA (data not shown) [47]. Maintaining functionality of the flagellar export apparatus/flagellar secretion system may provide a benefit to *motA* mutants over other flagellar mutants such as *flgR* or *flgS* that are unable to secrete proteins due to the lack of expression of the flagellar structure [50].

The current study of strain 81-176 demonstrated genetic variations beyond the expected phase variations, including the deletion of an intergenic region after limited passage *in vitro* as well as genetic variation comparable to that seen for differing lineages of NCTC11168 [38], [41]. Such genetic diversity amongst the *C. jejuni* population likely facilitates adaptation to the different environments encountered by this zoonotic human pathogen. While the re-sequencing data obtained herein, and that reported for NCTC11168, both demonstrate the genetic variation of *C. jejuni*, the current study links variation to a non-motile form with the appearance of colonial variants. Overall, the data shed light on the extent of genetic variation and instability seen for *C. jejuni* that complicates the investigation of this pathogen.

## Materials and Methods

### Bacterial strains and growth conditions

*C. jejuni* strains 81-176 (HS23/36) has been described previously [11], [23]. The *flaAflaB*::Cm mutant of 81-176 has also been previously described [25], [51]. The NMRC laboratory strain of 81-176 was originally derived from a volunteer fed the original strain at the Centers for Vaccine Development [19]. A stock of this strain was prepared at the Walter Reed Army Institute of Research Bioproduction Facility in 1994 under current Good Manufacturing Practices (cGMP) for use in clinical trials [20]. More recently the cGMP stock was plated for single colonies that were screened by immunoblot to identify colonies producing polysaccharide capsule [52] for other studies. One such encapsulated colony, 81-176/55, was expanded and used in this study (Figure 1A). For identification of variants, strains were plated at an appropriate dilution to observe ∼100 colonies/plate on Campylobacter Blood Agar (CBA; BBLCampy-BAP, BD, Sparks, MD). Otherwise, strains were routinely grown on Mueller-Hinton (MH) agar plates incubated in microaerobic atmosphere (85% N_2_, 10% CO_2_, 5% O_2_) at either 37°C or 42°C for 18 to 26 hours. Remel CVA plates (Thermo Fischer, Lenexa, KS), porcine brain heart infusion, MH supplemented with Blaser-Wang (BW; Oxoid Limited, Hampshire, England), and Brucella broth (BB) agar were additionally used for bacterial growth.

### Motility assays

Wet mounts were performed by examining viable bacteria, resuspended in PBS, by light microscopy. Single colonies picked from CBA plates were stabbed into semisoft motility agar, either MH 0.4% agar petri plates or BB 0.6% agar 24-well plates [53]. Growth occurred at 37°C under microaerobic conditions and plates were analyzed for motility by a plus/minus system after ≥24 hours.

### Transmission electron microscopy

For visualization of flagella, bacteria were grown in T25 biphasic cultures. The supernatant was removed, washed, and used as the sample for staining and imaging. The bacterial samples were negatively stained with uranyl acetate and images were taken on a JEM-100 CX II transmission electron microscope (JOEL Ltd., Peabody, MA) at the NMRC Research Services Directorate, Pathology Department (Silver Spring, MD).

### Immunoblot analyses

Whole cell preparations of *C. jejuni* were subjected to SDS-PAGE analysis on 12.5% tris-glycine gels, transferred to nitrocellulose, and blotted for flagellin and the phase variable regulators FlgR and FlgS using rabbit polyclonal antisera with a goat anti-rabbit secondary conjugated to HRP as previously described [25]. Blots were developed colorimeterically using BCIP/NBT (Pierce, Rockford, IL).

### Directed analysis of genetic content

Individual flagellar genes were PCR amplified from bacterial boil preps of non-motile variants using a proof-reading polymerase (Advantage HF2 polymerase, Clontech, Mountain View, CA) (see Table S2 for a list of genes and primers). PCR products were purified and sequenced on an Applied Biosystems 3100 sequencer (Foster City, CA). Sequences were aligned and compared to the WT sequence using the software program Sequencher 4.8 (Gene Codes Corporation, Ann Arbor, MI).

### Whole genome sequencing

DNA from 81-176/55 and four variants was isolated using phenol chloroform extraction and subjected to high-throughput genomic sequencing on the Hi-Seq platform (Illumina). Genomic sequencing libraries were constructed according to standard operating procedures at The Institute for Genome Sciences Genome Resource Center (http://www.igs.umaryland.edu/resources/grc/index.php). These methods have been utilized to generate genome data for over 1000 bacterial isolates (http://gscid.igs.umaryland.edu/).

### Sequence read mapping and annotation

Raw sequence reads were mapped to the reference genome, *C. jejuni* 81-176 (accession number CP000538.1), using Mira v3.4.0 [54]. Sequence and structural variants were identified and manually verified from the Mira output. The raw sequence data were deposited in the Sequence Read Archive (SRA) of the National Center for Biotechnology Information (Study accession # SRS463970).

### Validation of sequence differences by conventional sequencing

Following high-throughput genomic sequencing, regions of uncertainty were re-sequenced by conventional dideoxy sequencing methods. Forward and reverse sequencing primers (Table S2) were designed using the online Primer3 software [55]. Sequencing reactions were performed and assembled as described above. Similarly, the *motA* allele of variants was amplified by PCR (see Table S2 for primers used) and the amplicon was sequenced as described previously.

### Generation of *motA* insertional mutants

The *motA* gene was amplified from 81-176 such that BamHI restriction sites would flank the amplicon (see Table S2 for primers used). The PCR product was gel purified, ligated into BamHI-digested pBluescript, and transformed into DH5α. The kanamycin (Km) resistance gene *aph3* from plasmid pILL600 [56], [57] was cloned as a SmaI fragment into a unique NcoI site within *motA* located 466 bp from the ATG start of the 777 bp gene. The plasmid containing *motA::aph3* was electroporated with selection on Km into *C. jejuni* strain 81-176. Clones were screened with primers that bracketed the insertion point of the Km cassette to confirm a double crossover.

### Generation of a *pflA* mutant

A previously described pUC18 plasmid containing a large region of *pflA* with a kanamycin resistance cassette (*aph3*; previously described) inserted between the BclI sites within *pflA* (pRY301) [58] was purified from DH5α cells. The purified plasmid was electroporated into strain 81-176 and the resultant Km^R^ clone was screened for insertion by PCR using primers for *pflA* amplification (Table S2). 81-176*pflA*::*aph3* was verified as non-motile in motility agar.

### Phenotypic assessment of mutants

To ascertain the phenotype of 81-176 mutants, bacteria were harvested from MH plates and mixed with an 81-176 O variant. The mixture was diluted, plated on blood agar for colonial variants, and incubated for 3 days at 37°C. Single T and O colonies were picked and analyzed for antibiotic resistance by plating on MH with/without antibiotics and for motility in 0.6% semi-soft agar.

## Supporting Information

Figure S1 **Effects of mutation on MotA.** (A) Comparison of putative conserved residues between MotA of *E. c*oli and *C. jejuni*. Residues critical for functionality include Arg90, Glu98, Pro173, and Pro222; these are highlighted for *E. coli* with similar residues marked on the *C. jejuni* MotA as putatively having comparable roles. The alanine undergoing change to proline in Type B mutation is indicated in bold for *C. jejuni*. (B) Comparison of predicted transmembrane domains of *E. coli* and *C. jejuni* MotA. Prediction of transmembrane helices was performed using TMHMM Server v. 2.0 (http://www.cbs.dtu.dk/services/TMHMM/).
(TIF)

Table S1 **Summary of mutations identified in the five newly sequenced** ***C. jejuni*** **81-176 clones compared to the** ***C. jejuni*** **81-176 reference sequence.** The first column lists the gene/region affected by a mutation. The second column lists the function of the gene or intergenic region of the mutation. The third column specifies if the mutation occurred in a known GC tract. Column 4 indicates the consequence of the mutation on the predicted amino acid sequence. The sequence of mutations in intergenic regions is unknown. Column 5 indicates the type of mutation (SNP, single nucleotide polymorphism). Column 6 lists the nucleotide changes and base pair affected on the reference genome.
(XLSX)

Table S2 **This table lists the oligonucleotide primers used in this study.**
(DOCX)
