# Supplementary figures and images for: High Frequency, Spontaneous motA Mutations in Campylobacter jejuni Strain 81-176
Source: PLoS One. 2014 Feb 18;9(2):e88043. doi: 10.1371/journal.pone.0088043 (PMC3928116; doi:10.1371/journal.pone.0088043)

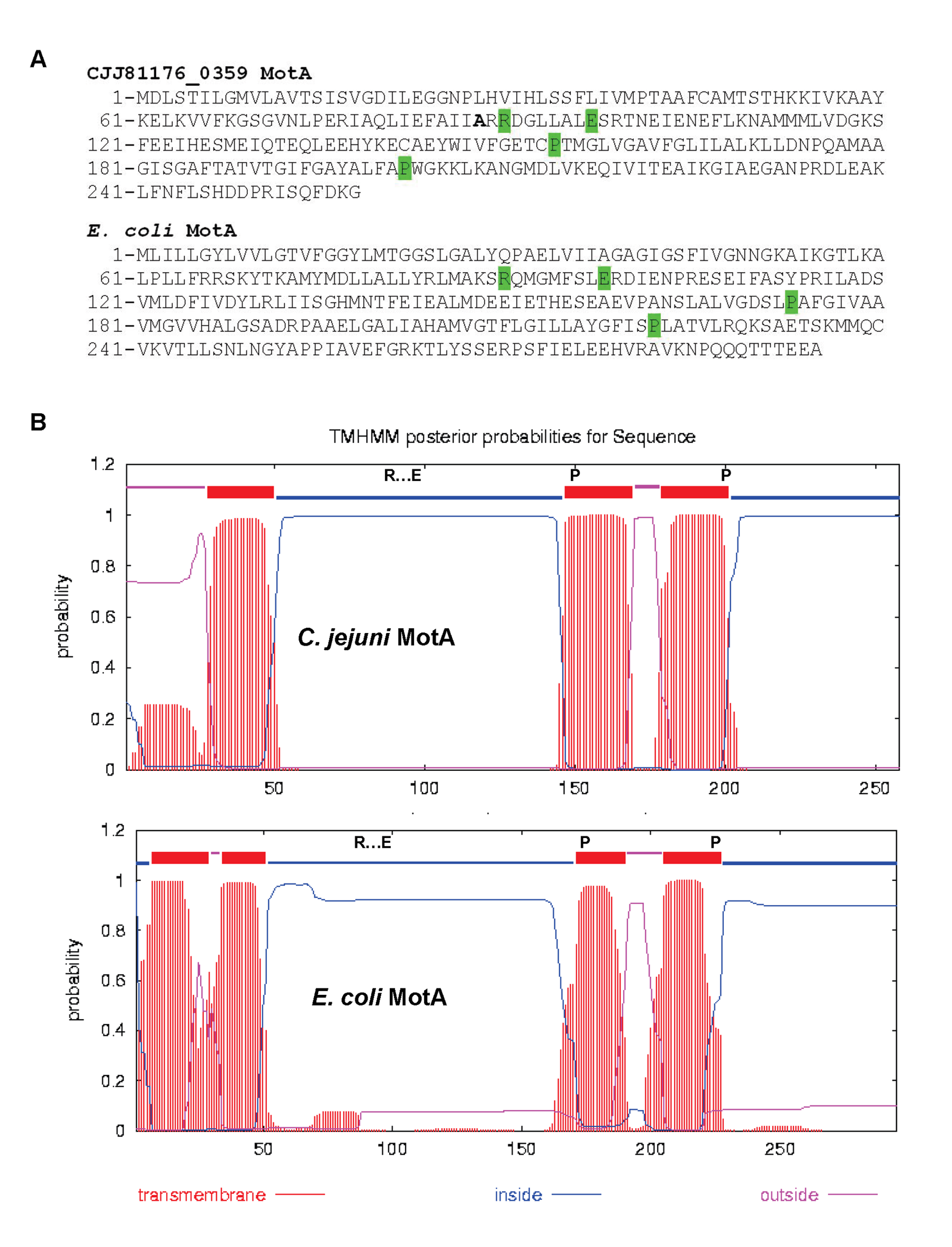

Supplement: Figure S1 — Effects of mutation on MotA. (A) Comparison of putative conserved residues between MotA of E. coli and C. jejuni. Residues critical for functionality include Arg90, Glu98, Pro173, and Pro222; these are highlighted for E. coli with similar residues marked on the C. jejuni MotA as putatively having comparable roles. The alanine undergoing change to proline in Type B mutation is indicated in bold for C. jejuni. (B) Comparison of predicted transmembrane domains of E. coli and C. jejuni MotA. Prediction of transmembrane helices was performed using TMHMM Server v. 2.0 (http://www.cbs.dtu.dk/services/TMHMM/). (TIF) [file pone.0088043.s001.tif]
