# Supplementary material for: High Frequency, Spontaneous motA Mutations in Campylobacter jejuni Strain 81-176
Source: PLoS One. 2014 Feb 18;9(2):e88043. doi: 10.1371/journal.pone.0088043 (PMC3928116; doi:10.1371/journal.pone.0088043)
Supplement: Table S2 — This table lists the oligonucleotide primers used in this study. (DOCX) [file pone.0088043.s003.docx]

**Table S2.** List of primers used in this study (restriction sites indicated with underlining).

| **Gene** | **Forward Sequencing Primer** | |  | **Reverse Sequencing Primer** | |  |
| --- | --- | --- | --- | --- | --- | --- |
| *pflA* |  | tgtgtttgtgtaagcacttttgtt |  |  | gcgtagctcaaattgatcaaactac |  |
| *flgP* |  | tgacgaggtggttagcattg |  |  | acattttcgctttgcgtcat |  |
| *flgQ* |  | agctttttgcccaagatgaa |  |  | ttttaaagcctcagtggcaga |  |
| *motA* |  | caagttcaagtatcgccaaaaa |  |  | tgccaaaagcaaacttagaaa |  |
| *cysK* |  | aacctagcgccaaagagtgt |  |  | aaggtgatagcatcagttttattgg |  |
|  |  |  |  |  |  |  |
| **Deletion** | **Forward Cloning Primer** | | | **Reverse Cloning Primer** | | |
| *motA* |  | gatcggatcccaagttcaagtatcgccaaaaa | |  | gatcggatcctgccaaaagcaaacttagaaa | |
